# Overtreatment and therapeutic nihilism in chronic epilepsy: a call to action!

**DOI:** 10.1186/s42494-026-00257-3

**Published:** 2026-06-01

**Authors:** Bernhard J. Steinhoff, John Paul Leach

**Affiliations:** 1 Epilepsiezentrum Kork, Landstrasse 1, 77694 Kehl-Kork, Germany; 2 Clinic of Neurology and Neurophysiology, University of Freiburg, Freiburg, Germany; 3 Angelini Pharma S.P.A., Viale Amelia 70, 00181 Rome, Italy

**Keywords:** Overtreatment, Therapeutic nihilism, Uncontrolled epilepsy, Patient management, Appropriateness, Treatment strategies

## Abstract

Despite the use of appropriately selected antiseizure medications (ASMs), seizure freedom remains elusive for some patients at each treatment line, and those failing two ASMs are then classified with drug-resistant epilepsy (DRE). Living with long-term uncontrolled epilepsy can lead to discouragement and disillusionment for both people with epilepsy and clinicians. In the context of potential treatment failure, maximizing seizure control while minimizing adverse events becomes even more critical, necessitating continual symptom surveillance and consideration of therapeutic trials of ASMs where appropriate. This requires truly individualized care, and in this pursuit, physicians will encounter the diametrically opposed challenges of overtreatment (or a burdensome drug load) and therapeutic nihilism (caused by missing consideration of treatment possibilities). This paper presents a detailed review of these issues and the influence of patient- and physician-related factors. Diagnostic and treatment strategies include ensuring accurate diagnosis and epilepsy classification for optimization of ASM treatment, considering the risk-to-benefit ratio when choosing treatment, encouraging treatment adherence, considering the initiation of alternative therapies, adjusting concomitant medications when needed, maintaining awareness of variations in patient characteristics, re-framing emotions effectively, and engaging in effective communication with patients and their families. All will have to be undertaken in conjunction with cognitive and psychological approaches. Seizure freedom is an important and worthwhile goal, and patients and families should be aware that increased therapeutic options available today can make seizure freedom possible even in the context of a challenging a priori prognosis or after previous ASM failures.

## Background

Epilepsy treatment usually requires long-term drug therapy to prevent relatively rare but burdensome symptoms, the chronic nature of which necessitates therapeutic adherence and ongoing support from an expert physician [1]. Uncontrolled seizures are a serious daily reality for those patients with epilepsy who are not achieving seizure freedom. Furthermore, as it is recognized that the chances of complete seizure control diminish with each antiseizure medication (ASM) failure, optimism may become more difficult to maintain for both patients and their healthcare providers, leading to nihilism and helplessness [2].

Persistent epilepsy symptoms require continual clinical follow-up, which in itself becomes a burden of illness, and the uncertainty of timing of paroxysmal can induce patient anxiety [3–5]. The costs of continued seizures are high: individuals not achieving seizure freedom encounter more stigma, suffer a poorer quality of life and a lack of social support, struggle with impaired cognition, and have an increased risk of comorbidities and death from accidents or sudden unexpected death in epilepsy (SUDEP) [1, 6–17]. Treatment options approved within the last 5 years offer potential benefit for people with epilepsy, and increased optimism for patients, caregivers, and physicians [18, 19]. Fears around seizures and their attendant risk, however, can present other errors in framing information relating to new treatment options.

Therapeutic nihilism may be defined as treatment reticence in patients or physicians and may result in underuse of potential efficient treatments [4, 20–22]. The obverse, overtreatment, is defined as unnecessary or excessive drug load leading to suboptimal management of the risk-to-benefit ratio of ASMs [4, 20–22]. For people with uncontrolled epilepsy, overtreatment can involve either unnecessary addition of ASMs or, for fear of further worsening symptoms after discontinuation, superfluous maintenance of the dose of an ineffective or poorly tolerated drug [20, 23]. Counterintuitively, therapeutic nihilism and overtreatment can co-exist simultaneously in the same patient: a patient can be overtreated with suboptimal ASMs while still lacking exposure to an optimal trial or dose of the most appropriate ASM.

In this article, we discuss the genesis of both overtreatment and therapeutic nihilism among individuals with uncontrolled seizures and present strategies to overcome these obstacles. This expert suggestion outlines current challenges encountered in clinical practice and is based on observations and data collected from people with epilepsy who were treated in clinical practices, often for decades.

## Clinical challenges in treating adult epilepsy

Clinicians may inadvertently err for many reasons when treating adults with epilepsy (Table 1). Inadequate communication is often the foundation, but internal biases, lack of insight or up to date knowledge, can combine to lead healthcare providers to engage in overtreatment or therapeutic nihilism [21, 24, 25]. Overtreatment compromises patient safety, reduces treatment adherence, impairs quality of life, is costly, and wastes resources [24, 25]. Nihilism results in treatment reticence, which reduces access to potentially more beneficial ASMs or the use of adequate doses.

**Table 1:** Ten Avoidable Errors Committed in Clinical Adult Epileptology

1. Failing to capture patient history precisely
2. Treating patients based on prior external reports instead of evaluating current symptoms and difficulties reported by patients, relatives, and eyewitnesses
3. Failing to personally review external diagnostic findings
4. Failing to conduct a personal physical examination
5. Lack of experienced physicians available in outpatient care
6. Failing to acknowledge and correct personal misjudgments
7. Overrating oneself as a physician
8. Rushing to treatment and not taking the necessary time to choose action
9. Leaving the patient uninformed
10. Avoiding continual communication with the patient during disease management

## Factors predisposing to overtreatment in the management of patients with epilepsy

Accurate diagnosis and classification of epilepsy is not always easy [26]. As a result, misdiagnosis is relatively common, thereby substantially reducing the likelihood that an inappropriate treatment will succeed [27, 28]. Overtreatment is more likely to occur after failure of multiple ASMs or when there is a clinical perception of a negative prognosis, a high seizure burden, or concern about the risk of injuries and SUDEP [29–31]. Patients’ unrealistic expectations or undue pressure from patient/caregiver can force the clinician into being seen to "do something." Healthcare providers may lose sight of the relative impact of seizures and ASMs, or having undue fear of inducing seizures by reducing or discontinuing ASMs. The frequency of overtreatment is difficult to document; however, it is observed in a variety of scenarios [20, 21].

## Factors predisposing to therapeutic nihilism in the management of patients with epilepsy

Numerous factors may influence the development of therapeutic nihilism, which can result from pessimism and disillusionment. Physician undertreatment and feelings of hopelessness may result from a variety of factors, including underestimation of seizure impact, disappointing experiences from previous ASM treatments, perceived skepticism of family/caregivers (especially while building trust during the transition from pediatric care to adult clinics), and fear of adverse effects from new and changing treatments [4]. Social effects of ongoing seizures may lead to lifestyle changes (such as medical retirement, or placement on social security benefits), causing patients or caregivers to withdraw pressure to modify treatment. Therapeutic nihilism is often influenced by the provider’s time pressures, where office visit time constraints may deter or diminish appropriate discussions regarding the potential benefits of further therapeutic strategies.

## Strategies to address the challenges of overtreatment and therapeutic nihilism

**Fig. 1:**
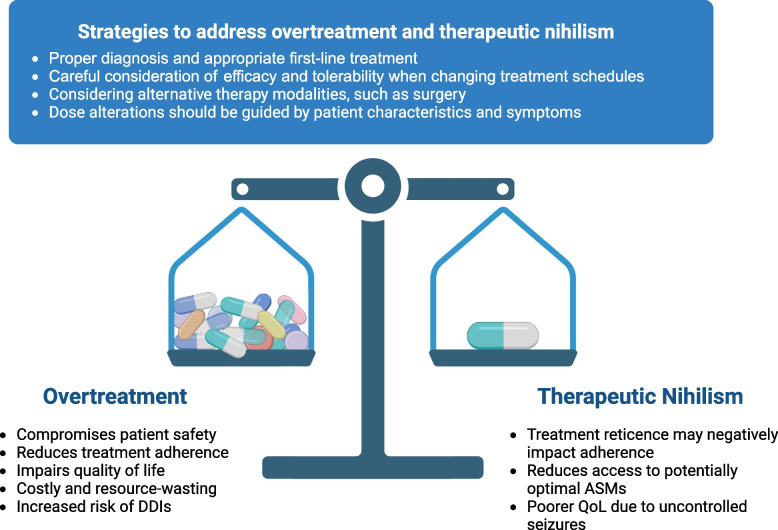
Strategies to Address Overtreatment and Therapeutic Nihilism. ASMs, antiseizure medications; DDIs, drug-drug interactions; QoL, quality of life

While there are concerns about the state of care for people living with epilepsy, therapeutic options are far from hopeless, and optimism is warranted. Diagnostic and treatment strategies can be adopted to combat the challenges of overtreatment and therapeutic nihilism (Fig. 1).

Proper diagnosis and appropriate choice of first-line treatment are critical components of successful patient management [27, 28]. Ensuring an accurate diagnosis and epilepsy classification of seizure type and syndrome enhances ASM efficacy and helps avoid the use of drugs that may provide incomplete efficacy or even worsen epilepsy. When changing treatment schedules, treatment adherence for patients and support by caregivers is encouraged through discussion of how efficacy and tolerability can be optimized, including how the impact of drug load may be managed. Consideration and discussion of the use of alternative therapy modalities, such as surgery, are vital but often neglected aspects of managing drug-refractory seizures [21]. Furthermore, dose alterations in individual patients should be guided by patient characteristics and symptoms rather than blind adherence to generally recommended doses or serum concentration ranges, honoring the concept of the individual’s own therapeutic range [20, 32].

At initial diagnosis, monotherapy should commence with the chosen first-line ASM at low doses, then the drug should be titrated appropriately to the target dose [20]. As the number of ASMs increases during management of uncontrolled seizures, the same principles should apply. Dose changes should be guided by clinical response, keeping in mind that modifying concomitant medications and reducing drug load may improve tolerability and effectiveness [21]. Clear assessment of efficacy following a recently added ASM is essential, and this will require some objective knowledge of seizure frequency before and after the new ASM was added. If the newly added ASM is ineffective at optimized doses, the drug should be withdrawn. The risk-to-benefit ratio of current and potential ASMs should be reassessed on a regular basis, and a “plan B” should be developed and shared with the patient as a backup in case “plan A” fails [21].

Cognitive and psychological strategies may also help providers mitigate or prevent overtreatment and therapeutic nihilism. Combination of nonanalytical and reflective reasoning may help in choosing the most effective treatment [24, 33]. Additionally, conscious identification and effective re-framing of emotions and an emphasis on the importance of the clinician’s own professional expertise may reduce physician biases [24]. These have been defined as Omission (Inaction) biases when a treating physician is fearful of the anticipated regret of an action taken (e.g., prescribing a treatment) and is overly concerned about potential blame if the treatment involves risk of potential harm; or Commission (Action) bias when the provider is fearful that inaction may be viewed as irresponsible [24]. Finally, physicians should maintain awareness of the effect of variations in patient characteristics on treatment decisions and response (e.g., race, sex, socioeconomic status, comorbidities) [24].

## Late successful response to ASMs in epilepsy treatment

In medical settings, there is a common misperception that achieving seizure freedom is highly unlikely following treatment failure with two ASMs [34], which is not universally borne out by the data. In a study at the Kork Epilepsy Center, 60% of patients achieved long-term seizure freedom, with 70% becoming seizure-free after using a maximum of five ASMs [35]. The emergence of newer ASMs for use in focal seizures and in some specific syndromes, such as Dravet syndrome, suggests that the outlook is continuing to improve [18, 36]. The established and emergent evidence indicates the treatment landscape for those with uncontrolled seizures is continuing to improve and may provide more grounds for optimism than previously believed. Re-educating patients and physicians with the latest data from epilepsy trials should help reduce therapeutic inertia and promote effective patient management.

Approximately half of those with newly diagnosed epilepsy reach seizure freedom with their first drug and, for the majority of the remaining patients, polytherapy will likely be initiated [37, 38]. Because adverse events (AEs) tend to increase with increasing drug load, determining the balance between efficacy and tolerability becomes critical; however, successful individualized treatment is still possible in patients even with a prior heavy drug load where there is adequate management of concomitant medications [3, 39].

When patients experience disabling or life-threatening seizures, early use of particularly efficacious and recently introduced ASMs, such as cenobamate or fenfluramine, may be viewed as critical. Indirect comparisons with older ASMs have shown that these drugs have demonstrated superior and sustained efficacy with similar tolerability, also conferring a meaningful reduction in mortality [18, 40, 41]. A systematic literature review of efficacy, safety, and tolerability data for ASM used in the treatment of focal seizures concluded that cenobamate was more effective than other ASMs such as perampanel, eslicarbazepine acetate, lacosamide, brivaracetam, and zonisamide [42]. Recent studies showed that ASMs such as adjunctive cenobamate effectively reduced seizures and allowed the reduction or elimination of polytherapy than what has been reported in trials of older ASMs [18, 39, 43, 44]. Similar outcomes may also be achievable with skillful use of other epilepsy treatment options [45]. However, these newest ASMs may be less frequently used due to a lack of awareness or knowledge regarding their improved efficacy compared to other ASMs [18].

## Conclusions

Epilepsy care, from a healthcare perspective, requires dedication, patience, and intelligence. While frequently rewarding, overtreatment and therapeutic nihilism are potential pitfalls during chronic management of treatment resistant epilepsy. To avoid these pitfalls, communication with patients and families is key. This includes outlining the potential risks and benefits of treatment changes and considering the patient’s unique epilepsy and medical history. More hazardous seizures, where risks of death or injury are increased, may justify more active management, and treatment escalation can be presented as a way of actively minimizing such risk. In patients especially prone to AEs or where other medications increase the risk of interactions, there must be allowance for a more measured approach to drug changes. Decisions on treatment amendment and adjustment will be more likely to be persistent where there is proactive patient and caregiver participation, requiring a holistic approach.

Nihilism should be confronted by increasing diagnostic and therapeutic opportunities that may offer significant optimism to patients and providers. Overcoming treatment nihilism requires awareness on all sides regarding the contemporary data around efficacy and the side effect profile of a heavy drug load. A realistic assessment for achieving seizure freedom is possible at all stages of the epilepsy journey, and conveying accurate seizure freedom rates will be vital in communicating the potential benefits of amended ASM treatment and motivating patients and caregivers. Anticipating and preventing overtreatment requires continued engagement and vigilance on the part of the clinician, critically considering the role and contribution of each ASM to the current treatment regimen. Treatment reduction need not always follow a “last in–first out” approach, as sometimes AEs related to a specific interaction can limit dose optimization of the newest add-on. In such cases, minimizing pharmacokinetic and pharmacodynamic interactions might allow optimization of the baseline ASM. Seizure reduction or freedom for patients who were previously refractory to multiple ASMs, may allow the patient to reduce or eliminate concomitant medications, resulting in an even greater improvement in quality of life.

## Data Availability

No datasets were generated or analysed during the current study.

## Abbreviations

AEs: Adverse events
ASMs: Antiseizure medications
DDIs: Drug-drug interactions
DRE: Drug-resistant epilepsy
SUDEP: Sudden unexpected death in epilepsy
QoL: Quality of life
